# Genetic Diversity of the Hepatitis B Virus Subgenotypes in Brazil

**DOI:** 10.3390/v11090860

**Published:** 2019-09-15

**Authors:** Barbara V. Lago, Marcia P. do Espirito-Santo, Vanessa D. Costa, Vanessa A. Marques, Livia M. Villar, Lia L. Lewis-Ximenez, Elisabeth Lampe, Francisco C. A. Mello

**Affiliations:** 1 Laboratório de Hepatites Virais, Instituto Oswaldo Cruz, FIOCRUZ, Rio de Janeiro, RJ 21040-900, Brazil; v.duarte391@gmail.com (V.D.C.); vmarques@ioc.fiocruz.br (V.A.M.); liviafiocruz@gmail.com (L.M.V.); lialewis.fiocruz@gmail.com (L.L.L.-X.); elampe@ioc.fiocruz.br (E.L.); 2 Instituto de Tecnologia em Imunobiológicos (Bio-Manguinhos), FIOCRUZ, Rio de Janeiro, RJ 21040-900, Brazil; paschoalms@gmail.com

**Keywords:** hepatitis B virus, subgenotypes, Brazil

## Abstract

Hepatitis B virus (HBV) subgenotypes may be related to clinical outcomes and response to antiviral therapy. Most Brazilian studies on HBV subgenotypes are restricted to some regions and to specific population groups. Here, we provide an insight about genetic diversity of HBV subgenotypes in 321 serum samples from all five geographical regions, providing a representative overview of their circulation among chronic carriers. Overall, HBV/A1 was the most prevalent subgenotype, being found as the major one in all regions except in South Brazil. Among HBV/D samples, subgenotype D3 was the most prevalent, found in 51.5%, followed by D2 (27.3%) and D4 (21.2%). D2 and D3 were the most prevalent subgenotypes in South region, with high similarity with European strains. D4 was found in North and Northeast region and clustered with strains from Cape Verde and India. For HBV/F, the most frequent subgenotype was F2 (84.1%), followed by F4 (10.1%) and F1 (5.8%), closely related with strains from Venezuela, Argentina and Chile, respectively. Phylogeographic analyses were performed using an HBV full-length genome obtained from samples infected with genotypes rarely found in Brazil (B, C, and E). According to Bayesian inference, HBV/B2 and HBV/C2 were probably introduced in Brazil through China, and HBV/E from Guinea, all of them mostly linked to recent events of human migration. In conclusion, this study provided a comprehensive overview of the current circulation of HBV subgenotypes in Brazil. Our findings might contribute to a better understand of the dynamics of viral variants, to establish a permanent molecular surveillance on the introduction and dispersion patterns of new strains and, thus, to support public policies to control HBV dissemination in Brazil.

## 1. Introduction

Despite the implementation of a successful vaccine in several countries, eradication of hepatitis B virus (HBV) is still a challenge. It is estimated that 2 billion people have been exposed to HBV worldwide and 240 million are currently at risk of developing cirrhosis and hepatocellular carcinoma due to chronic infection. Chronic carriers are the main reservoirs of HBV and probably act as a major driven force on HBV evolution. During a long-lasting chronic infection, different selective pressures, as immune system and/or antiviral therapy, shape viral populations. HBV evolution occurs inter and intra-hosts and is punctuated by the geographic distribution of HBV variants. HBV evolution rate has been estimated varying from 2.2 × 10^−6^ to 7.7 × 10^−4^ nucleotide substitutions/site/year [1,2,3,4,5]. These discrepancies may be due to differences in the methodology employed for HBV evolution rate calculations. Whereas long-term studies employed internal node calibrations on phylogenetic trees using conserved regions of the HBV genome [3], the short-term studies have been performed on HBV evolution within a single patient or are based on family history or pedigree [2,5,6,7]. 

Due to its unique life cycle, in which an error-prone reverse transcriptase is employed for genome replication, HBV presents a large genetic variability, resulting in at least 9 genotypes (A–I), almost 40 subgenotypes, several recombinants, clades and quasispecies [8]. Genotype A is currently divided in 4 subgenotypes, named A1–A4. Subgenotypes A1, A4, and quasi-subgenotype A3 are endemic in Africa, while A2 prevails in Europe and North America. Genotypes B and C are prevalent in East Asia, Indonesia and Oceania. Genotype B have been divided into 6 subgenotypes: B1, B2, B4–B6, and quasi-subgenotype B3, whereas genotype C, the oldest HBV genotype, has the highest number of subgenotypes: C1, quasi-subgenotype C2, C3–C16. Genotype D has a worldwide distribution and is currently divided in 9 subgenotypes/recombino-subgenotypes: D1–D9. Genotype E is found almost exclusively in West Africa and is not divided into subgenotypes. Its low genetic diversity is consistent to a short evolutionary history in humans. Genotype F is classified into 4 subgenotypes, F1–F4, and are more frequent in Amerindian populations of Central and South America. This geographic distribution is also shared by genotype H, which is genetically related to genotype F. Genotype G does not have a distinct geographic distribution and has been related to a specific group as men who have sex with men in samples from Europe, USA, Mexico, Brazil and Japan. Genotype I was first reported in a single sequence from Vietnam. Subsequently, sequences found in Laos, India and China allowed the classification into two subgenotypes: I1 and I2 [8,9,10,11,12,13]. More recently, a putative subgenotype J was proposed from a single sample found in Japan [14].

Genotypes A, D and F are the most prevalent in Brazil, reflecting Brazilian population origins, descendant mainly from African slaves, European colonizers and native Amerindians [15,16,17]. Moreover, the number of immigrants is increasing considerably, leading to demographic changes and introduction of foreign viral variants.

Recently, a large-scale study with more than one thousand sample spanning virtually all Brazilian States and hundreds of cities countrywide, revealed a markedly difference in genotype distribution in the distinct geographic regions. While HBV/A was the most prevalent in the North and Northeast regions, HBV/D in South region, the HBV/F was the second most prevalent genotype in the Northeast region. Genotypes B, C, E and G were found in a minor proportion, so that in total, seven HBV genotypes were found circulating in Brazil [18]. There are increasing evidences that HBV subgenotypes may be related to clinical outcomes, as progression to hepatocarcinoma, and the response to antiviral therapy [9,12,13]. Despite several studies on HBV subgenotypes have been performed in Brazil, most are restricted to some regions and to specific population groups [17,19,20]. In this study, we analyzed the genetic diversity of HBV subgenotypes from all five geographical regions, providing a representative overview of subgenotypes circulation among chronic carriers. Moreover, Bayesian evolutionary analyses were conducted for the subgenotypes rarely found in Brazil to estimate the most probable dissemination routes.

## 2. Materials and Methods

### 2.1. Study Population

Serum samples from 321 chronic hepatitis B carriers (HBsAg positive and HBV DNA load > 200 IU/mL) were included in this study. All samples were collected between 2013–2015, from capitals and countryside cities of the five Brazilian geographical regions, and previously genotyped by “INNO-LiPA HBV Genotyping assay” [18]. Except for HBV/F, the proportion of samples from each genotype selected for subgenotype characterization was similar to the proportion of genotype distributions observed in the correspondent regions, as stated in the nationwide multicenter study representing all Brazilian regions [18]. Therefore, 177 samples of genotype A (55.1%), 66 samples of genotype D (20.6%) and 69 of genotype F (21.4%) were included in this study. All samples from HBV/A, /D, and /F were selected using the random sample selection tool in our Excel datasheet. In addition, 9 samples (2.8%) from the genotypes rarely found in Brazil were also selected: B (*n* = 1), C (*n* = 2), E (*n* = 3) and G (*n* = 3). Genotype F sampling was overrepresented as a way of providing molecular data about this less studied genotype as required by other researchers [8,12].

### 2.2. Ethics Statement

The study was approved by the Ethical Committee of the Oswaldo Cruz Foundation (FIOCRUZ) in December 16th, 2013 under protocol number 495.687 and it is in agreement to the ethical guidelines of the 1975 Declaration of Helsinki.

### 2.3. Viral DNA Extraction

HBV DNA was isolated from serum samples using the Biopur Mini Spin Viral DNA Extraction Kit (Biometrix Diagnostica, Paraná, Brazil) according to the manufacturer’s instructions. HBV DNA was stored for later use for genome amplification and direct nucleotide sequencing followed by phylogenetic analyses.

### 2.4. HBV DNA Sequencing and Subgenotype Characterization

All 321 samples were submitted to PCR amplification of partial S/Pol genes containing 805 bp, using primers and thermal cycling conditions as described by Mallory et al. (2011) [21]. Genotypes rarely found in Brazil as B (*n* = 1), C (*n* = 2), E (*n* = 1) and G (*n* = 1) were submitted to full-length genome amplification and sequencing (~3200 bp). Another 14 Brazilian samples from different HBV genotypes endemic in Brazil were also submitted to full-length amplification, as described by Günther et al. (1995) [22], in order to verify the accuracy of HBV subgenotypic classification of the partial S/Pol fragment (~800 bp) compared to the full-length genome. Then, phylogenetic analyses for both complete and partial sequences were conducted with additional 65 sequences from all HBV subgenotypes available in GenBank (see list and origin of all reference sequences used in this study in Table S1). Multiple sequence alignment was performed by using Clustal W program implemented in MEGA software version 7.0 [23]. 

Phylogenetic analysis was carried out using the maximum likelihood method, bootstrap resampling test with 1000 replicates. The dispersal pattern of the rare/unusual genotypes as B, C and E was accessed by Bayesian Inference using the Bayesian Markov Chain Monte Carlo (MCMC) statistical framework implemented in the BEAST v1.10 package [24] under GTR (General Time Reversible) + G + I, which was selected as the best-fit model. Phylogeographic datasets were performed according to the following criteria: non-recombinant human full genome sequences with known country and collection date, whose nucleotide sequences did not present any insertion. The number of sequences from the same locality was proportionally adjusted in order to avoid bias. MCMC analysis was run for 1 + E08 generations. Calculation of the effective sample size (ESS) was performed using TRACER v1.7. All parameters showed ESS values >200 and their uncertainties were reflected in the 95% Highest Posterior Density intervals. The maximum clade credibility was visualized with FigTree v1.4.2 program.

## 3. Results

Nucleotide sequences of the partial S/Pol fragment (805 bp) were obtained from 321 Brazilian HBV positive samples. Full-length genome sequences (~3200 bp) were successfully obtained from the rare genotypes B, C and E, as well as from other 14 samples of genotypes A, D and F circulating in Brazil. Phylogenetic analysis was performed along with 65 sequences from all HBV subgenotypes (complete genomes) available in GenBank. 

**Figure 1:**
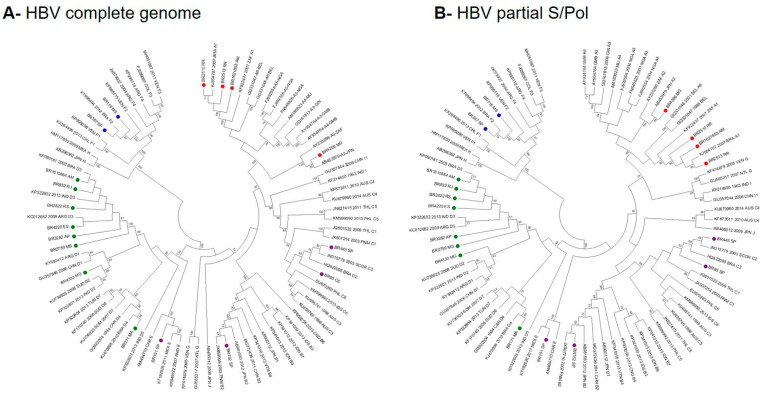
Comparison of the full-length and partial S/pol phylogenetic trees topologies. Red dots: HBV/A; purple dots: HBV/B, C and E; green dots: HBV/D; blue dots: HBV/F genotypes/subgenotypes.

A comparative analysis of the topologies of full-length and partial S/pol phylogenetic trees was performed aiming to verify the accuracy of HBV subgenotypic classification using partial S/Pol fragment. The Brazilian HBV strains clustered with the sequences of reference subgenotypes with high bootstrap value (> 80%) in both full-length (Figure 1A) and partial S/Pols phylogenetic analysis (Figure 1B). The classification of HBV strains into subgenotypes was the same in both phylogenetic trees. Since no significant differences between trees topologies were observed, the analyses of HBV subgenotype prevalence and phylogenetic relationships were based on the partial S/Pol fragment. 

**Figure 2:**
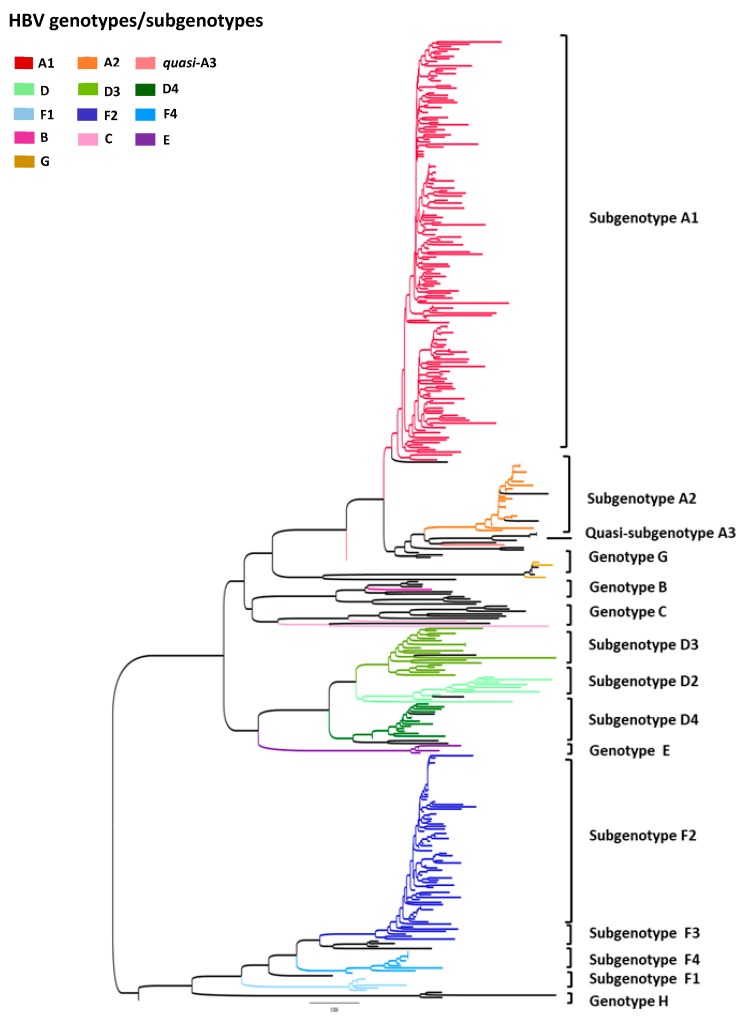
Maximum likelihood phylogenetic tree of all samples enrolled in this study (*n* = 321). Each genotype/subgenotype clade is represented by colours discriminated in the legend and taxa used as reference sequences are displayed in black.

**Figure 3:**
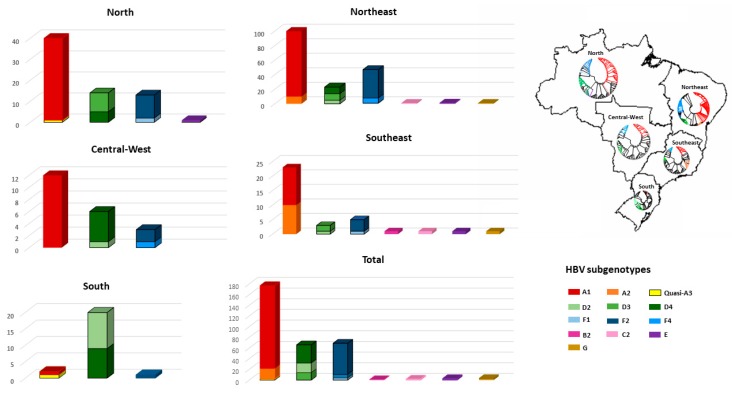
Distribution of HBV genotypes according to geographic regions. The phylogenetic trees in Brazilian map reflects the HBV subgenotypic diversity in each region. Taxa represented in black are reference sequences retrieved from Genbank.

Phylogenetic analyses of partial S/Pol gene sequences (805 bp) from all 321 HBV-DNA positive samples revealed that, overall, HBV/A1 was the most prevalent subgenotype (Figure 2). From HBV/A samples selected for subgenotyping (*n* = 177), subgenotype A1 was detected in 87.6% and A2 in 11.3%. Two samples (1.1%) belonging to HBV/A could not be subgenotyped, clustering close to sequences for the quasi-subgenotype A3, however without a strong bootstrap support (Figure 2 and Figure 3). Among HBV/D samples (*n* = 66), subgenotype D3 was the most prevalent, found in 51.5%, followed by D2 (27.3%) and D4 (21.2%). For HBV/F (*n* = 69), the most frequent subgenotype was F2, in 84.1% samples, followed by F4 (10.1%) and F1 (5.8%). 

HBV/B full-length genome (*n* = 1) was classified as subgenotype B2 and HBV/C (*n* = 2) as *quasi*-subgenotype C2. One genotype E full-length sequence was obtained and genetically analyzed. Unfortunately, genotype G could not be successfully full-length amplified and reamplification was not possible due to sample volume limitation. Figure 2 show the maximum likelihood phylogenetic tree of partial S/Pol gene sequences (805 bp) from all 321 HBV samples (subgenotype-specific clades are indicated by colours).

**Figure 4:**
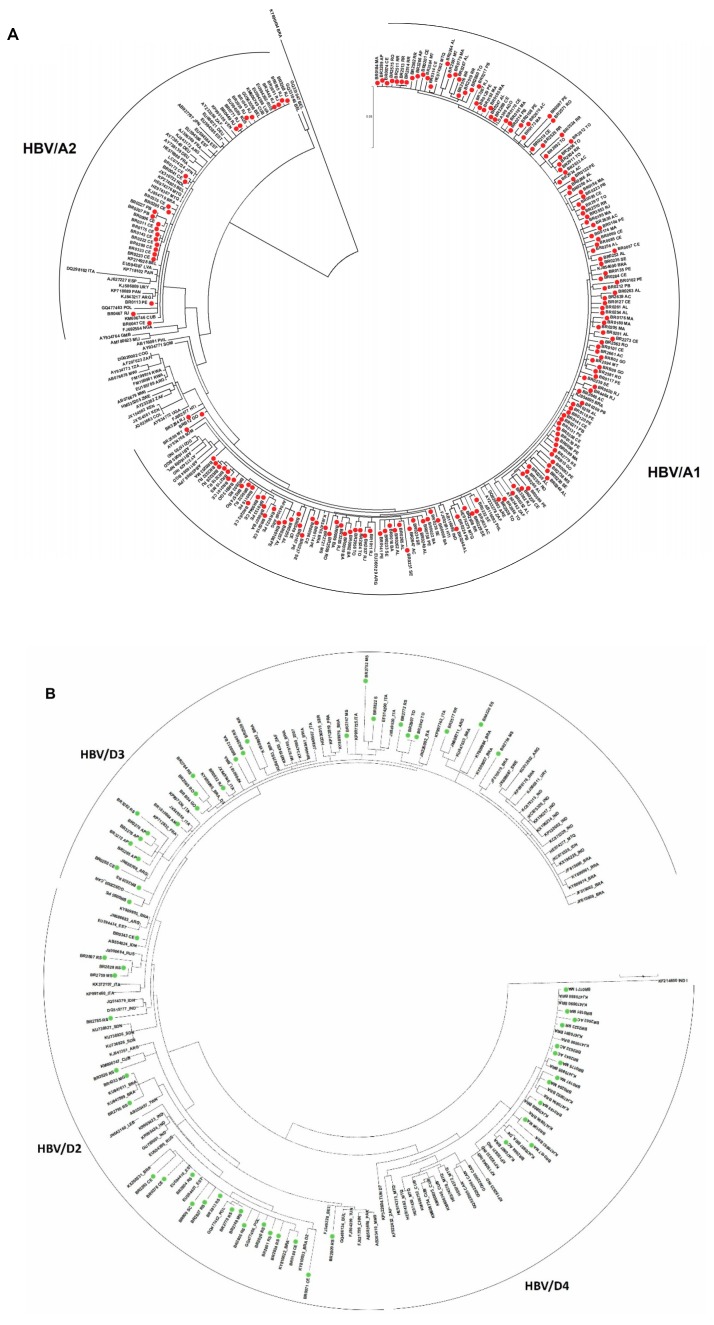

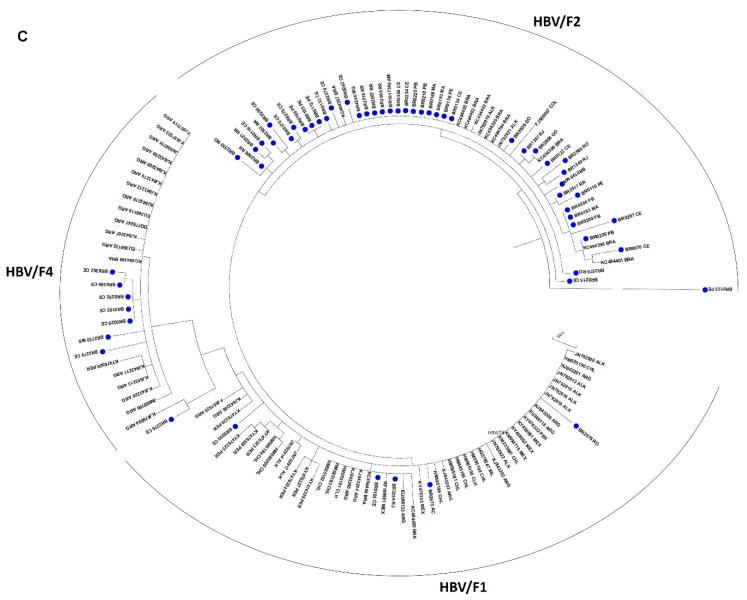
Phylogenetic analysis based on HBV partial S/Pol nucleotide sequences. Maximum likelihood phylogenetic trees for each genotype were constructed using (**A**) 177 HBV/A; (**B**) 66 HBV/D, and (**C**) 69 HBV/F sequences determined in this study and reference sequences representing all subgenotypes.

HBV subgenotype distribution according to region is detailed in Figure 3. HBV/A1 was the most prevalent in all but the Southern region, with all Brazilian isolates clustering in the Asia-American clade (Figure 4A). In the North, A1 accounts for 57.3% of HBV isolates. HBV/A2 was detected in Northeast (5.8%) and Southeast (25.7%). HBV/D2 (47.8%) and D3 (39.1%) were the most prevalent subgenotypes in South, presenting high genetic identity with European samples. HBV/D4 was found in North and Northeast and clustered with strains from Brazil, Cape Verde and India. As identified by Lampe and colleagues, most of HBV/F samples were observed in Northeast. From all Brazilian HBV/F isolates, HBV/F2 was the most frequent (84.5%), followed by F4 (10.1%) and F1 (5.8%), presenting genetic relatedness with strains from Venezuela, Argentina and Chile, respectively. Genotypes B, C, E and G were found mainly cases in the Southeast region (Figure 3).

**Figure 5:**
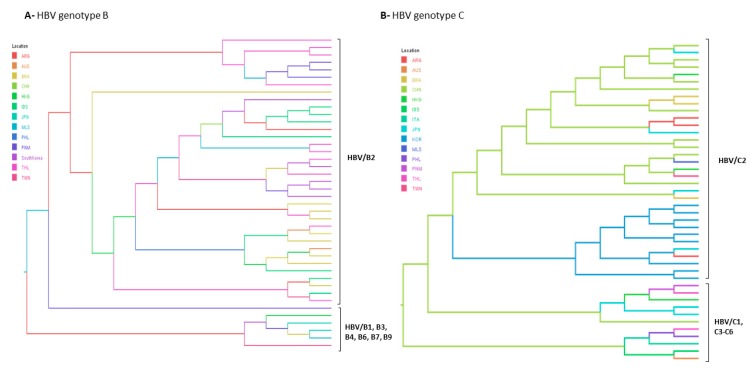

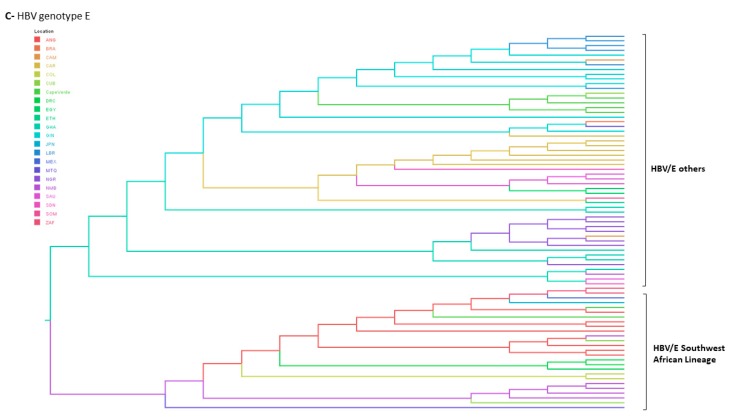
Bayesian maximum clade credibility tree of (**A**) HBV/B, (**B**) HBV/C and (**C**) HBV/E full length genome sequences. B1–B9 and C2–C6 represent HBV subgenotypes within genotypes B and C respectively. HBV/E tree is composed by two branches corresponding to the Southweast African lineage countries (Angola, Namibia and Democratic Republic of the Congo) and the other HBV/E sequences found in other African countries. Branches are coloured according to probable country of origin represented by the following abbreviations: ANG: Angola; ARG: Argentina; BRA: Brazil; CAM: Cameroon; CAR: Central African Republic; CHN: China; CAN: Canada; COL: Colombia; CUB: Cuba; CPV: Cape Verde; DRC: Democratic Republic of the Congo; EGY: Egypt, ETH: Ethiopia; GHA: Ghana; GIN: Guinea; HKG: Hong Kong; IDN: Indonesia; JPN: Japan; LBR: Liberia; MEX: Mexico; MTQ: Martinique; NGR: Nigeria; NMB: Namibia; PNM: Panama; SAU: Saudi Arabia; SDN: Sudan; SOM: Somalia; THL: Thailand; VTN: Viet Nam; ZAF: South Africa.

Bayesian evolutionary analyses were conducted for the subgenotypes rarely found in Brazil as B2, C2 and E. Maximum clade credibility trees displayed in Figure 5 shows the most probable country of origin and dissemination routes of these genotypes to Brazil. According to Bayesian Inference, HBV/B2 and HBV/C2 were likely introduced in Brazil from China. Our results confirmed that both HBV/B2 and C2 would have originated in China, as previously proposed [3,25]. HBV/E clustered with samples from Liberia, Ghana and Guinea and it was probably introduced in Brazil from Guinea.

## 4. Discussion

Recently, a nationwide, large-scale survey on the prevalence and geographic distribution of HBV genotypes in Brazil was published by our group [18]. The present study is a complementation of the previous data, aiming to elucidate in detail the genetic variability of HBV subgenotypes across Brazil, as well as to investigate the introduction and dispersion patterns of foreign viral variants. Phylogenetic analysis of 321 HBV isolates revealed the presence of three HBV/A (A1, A2, quasi-A3), three HBV/D (D2-D4) and three HBV/F (F1, F2 and F4) subgenotypes circulating endemically in Brazil. Genotypes B, C, E and G were mostly linked to travelers or immigrants. 

As genotype A has been found worldwide, it has been suggested that it has emerged in Africa [4,26,27] and after a long evolutionary process and differentiation events, it has accumulated enough genetic variability to permit the classification in different subgenotypes before and after spreading to other continents [28]. Previous studies proposed that HBV/A1 was first introduced in Brazil by the slave trade from 16th to 19th centuries [15,17]. In this study, HBV/A1 was by far the most prevalent subgenotype, accounting for 87.6% of HBV/A samples, while A2 was detected in 11.3%. These findings corroborated with previous observations in which HBV/A1 has been found at a frequency about ten times higher than A2 in Brazil [15,16,29,30]. Although not presenting a monophyletic origin —which indicate multiple introduction events over time— all Brazilian HBV/A1 isolates clustered in the ‘Asia-American’ instead of the ‘African’ clade. This observation reinforces the suggestion that HBV/A1 isolates were carried to Brazil by an alternative route, possibly being imported from East Africa or Asia by merchants in the middle of the 19th century [30]. Further studies enrolling samples from East African countries as Mozambique would shed a light on HBV/A1 dispersion patterns between Africa and Brazil.

Assuming an intermediate mutation rate of 2 × 10^−5^ s/s/y [1,26], it was suggested that HBV/A1 and A2 have diverged more than 2000 years ago, with HBV/A2 isolates being endemic in Europe and countries with European colonization [8,26]. It is known that the main subgenotypes circulating in Europe are HBV/A2 in the North-western countries and HBV/D1, D2 and D3 in the South-eastern European and Mediterranean countries [31,32]. In this study, HBV/A2 isolates clustered with sequences from distinct European countries such as Germany, Belgium, Russia and Poland and are phylogenetically divided into several branches, with no evidence of monophyly (intragroup and overall mean genetic distance 0.007 ± 0.001).

Two HBV/A sequences from this study (one from North and the other from South region) clustered close to the recently described quasi-subgenotype A3, that comprises sequences from the former A3, A4 and A5 subgenotypes [12]. Unfortunately, some ambiguities found in these sequences could not be resolved due to volume limitation for resequencing, so more sequences are needed to confirm the circulation of quasi-subgenotype A3 in Brazil.

Genotype D has been found in all five Brazilian geographic regions, being the second most prevalent genotype [16,18]. In this study, we observed a marked prevalence of HBV/D2 and D3 in South region (87.0%). These findings were in agreement with other studies conducted in South Brazil, where these subgenotypes accounted for 57–100% of viral isolates [33,34,35,36,37]. Phylogenetic analysis revealed that HBV/D2 and D3 isolates were genetically related to sequences from East Europe and Italy, respectively. HBV/D3 is highly prevalent in Italy [31,38] and have been described in populations of Italian ancestry in Brazil [20,36,39,40,41] and Argentina [42]. In our study, all Brazilian D3 clustered together, suggesting closer ancestral relationships (intragroup divergence of 0.005 ± 0.001, versus 0.007 ± 0.001 when compared to international sequences). However, a study enrolling full-genome analysis of HBV/D3 from Brazilian Amazon Region published by Spitz and colleagues [43] did not find a close relatedness between the Brazilian and European D3 sequences. However, this might be due to the limited number of HBV/D3 full-length genomes assigned to Italy in Genbank that hindered more robust phylogenetic analyses. Despite this, combined evidence as historical background, partial HBV/D3 sequencing, and Y-chromosome heritage pointed out Italy as the most plausible source of HBV/D3 circulating in Brazil [36,39,41,44].

While HBV/D3 may be related to Italian settlement in South region in the early 1900s, the detection of HBV/D4 in Brazilian North and Northeast region seems to be linked to the forced migration of Africans to Brazil. Despite virtually confined to Africa, HBV/D4 has been found in the islands of Cuba, Haiti and Martinique, where, as HBV/A1, its introduction has been linked to slave trade [28,45]. Here, HBV/D4 was detected in 21.2% of HBV/D samples, all of them from North and Northeast Brazil. This finding is in agreement with previous studies conducted in rural populations of the same Brazilian regions, where HBV/D4 have been found in a variable frequency, from 2.5 to 24% [29,46,47]. The relatedness of the Brazilian strains thus suggests single/few introductions of HBV/D4 in North/Northeast Brazil. Phylogenetic analyses showed that the Brazilian HBV/D4 formed a distinct clade, with few sequences from East Africa, Cape Verde and from the region of Tripura, India. As Brazil, India also faced successive waves of colonization, and multiple episodes of human migration [48]. It is possible that, due to the geographical proximity of Tripura with the Portuguese colony of Hughli in India, HBV/D4 isolates would have been introduced in Brazil from Asia or East Africa by the Transatlantic trade, as stated for the Asian-American clade of subgenotype A1. 

Latin America is the most plausible origin of HBV/F, where it circulates since the pre-Columbian times [49]. HBV/F subgenotypes are highly dispersed in South, Central America and in the native population of Alaska, being rarely found in other parts of the globe [33,50,51,52,53,54].

Although South American countries present a marked predominance of HBV/F, several historical processes shaped Brazil as an exception [18,36,54]. HBV/F is the third most prevalent genotype circulating in Brazil, with a remarkably prevalence in the Northeast, region with a high interchange with the indigenous population from Amazon region [16,18]. In this study, the most frequent HBV/F subgenotype was F2 (84.1%), followed by F4 (10.1%) and F1 (5.8%). A very similar prevalence was found by Mello and colleagues [54] in a survey on the phylogeography of HBV/F in Brazil, in which HBV/F2 was suggested as being the oldest HBV/F subgenotype, thus representing the original native HBV of Brazil [54]. HBV/F2 have been described in variable proportions across Brazil [16,39,55,56]. Here, HBV/F2 was mostly found in North and Northeast displaying high similarity with other Brazilian sequences and with sequences from Venezuela. HBV/F4 and F1 were related to strains from Argentina and Chile, respectively.

Although Brazilian ethnical background seems to explain the genetic variability of HBV strains, viral variants of unusual/rare subgenotypes were mostly associated to immigrants or travelers. In this study, one HBV/B2 sample was identified in an Asian patient living in Southeast Brazil, and clustered with HBV/B2 sequences from China and Panama. To our knowledge, a complete genome of subgenotype B2 has not been characterized in Brazil before. Although HBV/B has been previously reported in a Chinese female who had moved to Northeast Brazil [57] and in patients from an Asian community in South region [58], no information regarding subgenotype variability was provided.

Phylogeographic analyses revealed that HBV/B2 would have originated in China and have been introduced in Brazil and other Latin America countries by China/Hong Kong. These findings are in agreement with the fact that Brazil, Argentina and Panama were the main destinations for Chinese immigrants from the 1960s onwards. Moreover, Brazil was the fourth country with the largest number of Chinese migrants in the XXI century, of whom 80.7% settled in Southeast region. This fact may be related to the expansion in commercial relations between Latin America and China, with an active Brazilian participation [59]. 

The two HBV/C isolates from this study were classified as quasi-subgenotype C2, one from Southeast and one from Northeast regions. According to phylogeographic analyses, China was the most plausible source of this subgenotype. HBV/C2 had been previously described in people of Japanese and Chinese ancestry from South and Southeast Brazil [20,37,58]. Despite HBV/B and C are widely found in the Asian region with an expressive number of HBV chronic carriers (revised in [8,60]), these genotypes are rarely detected out of this continent. In Brazil, most reports of genotypes B and C have been linked to Asian communities and probably have been restricted to these groups due to cultural habits and/or intrafamilial transmission routes [58,61].

It has been established that genotype C has a higher propensity to fix mutations compared to genotype B, and for this reason have been linked to severe liver disease and hepatocarcinogenesis [8]. These findings highlight the need to manage the dispersion of HBV/C in Brazil, since its spread may seriously impact the public health.

HBV/E is largely spread in West Africa but its low genetic variability pointed out to a recent evolutionary history in humans [28,62,63,64]. Despite the three centuries of slave trade from Africa to Americas and the evidences of the contribution of forced migration in the spread of other HBV genotypes, HBV/E has not been reported in the Americas, except in people who maintained relations with Africa [17,19,37]. In this study, HBV/E was characterized in samples from North, Northeast and Southeast, in individuals whose names indicate African ancestry. In Brazil, HBV/E was previously found in a patient from Guinea-Bissau living in South region [37], in an Angolan individual living in an Afro community from Central Brazil [17] and in our multicentric previous study, where HBV/E was detected in sporadic cases in all Brazilian regions, except the South [18]. 

Phylogenetic analysis of HBV/E partial sequences indicates independent introductions of this genotype in Brazil. From all three HBV/E sequences from this study, two clustered in the Southwest African Lineage (SWA), that comprises samples from the Democratic Republic of Congo, Namibia and Angola, countries located in the West African coast, that historically were suppliers of slave labor to Brazil [62].

Phylogeographic analyses were performed with one HBV/E complete genome that was successfully amplified. To our knowledge, no complete genome of HBV/E has been characterized in Brazil before. This isolate did not fit in the SWA and may not represent the consensus of HBV/E strains circulating in Brazil. This analysis revealed that the most recent common ancestor of this strain was possibly introduced in Brazil through Guinea, probably linked to the recent episodes of human migration from Africa. However, due to the remarkably differences in the HBV/E sampled in Brazil, these results cannot be extrapolated to other HBV/E isolates.

Genotype G could not be successfully amplified in its full length. Nevertheless, phylogenetic analysis of the three HBV/G partial sequences revealed multiple introductions through distinct origins. HBV/G is a cosmopolitan genotype that have been linked to sexual transmission by men who have sex with men worldwide [8,60]. HBV/G is commonly found in coinfection with other genotypes, however, few cases of mono-infection have been described [65,66], as the chronic carriers enrolled in this study. 

Brazil is a continental country with expressive ethnic diversity shaped by several historical events. The genetic variability of HBV expressed in the different subgenotypes reflects the Brazilian ethnic background that seems to be allied with the distinct waves of migration to and inside Brazil. In addition, recent episodes of Arab and Asian migration have contributed to ethno-cultural enrichment of the Brazilian population as well as to the dispersion of pathogens. 

In conclusion, a comprehensive overview of the current situation of HBV subgenotypes circulation among chronic carriers in all Brazilian regions is addressed in this study. These data may contribute to epidemiological surveillance of HBV isolates circulating in Brazil, introduction of new strains as subgenotypes B2, quasi-C2 and E and its dispersion patterns, mostly linked to the recent events of human migration. Combined efforts of research groups and the Brazilian Ministry of Health are needed to better understand the dynamics of viral variants, to establish a permanent molecular surveillance to monitor the introduction of new strains and, thus, to interrupt the chain of HBV dissemination in Brazil.

## Supplementary Materials

The following are available online at https://www.mdpi.com/1999-4915/11/9/860/s1. Table S1: Sequences used in HBV complete genome phylogenetic analyses.
