# Supplementary material for: Genetic Diversity of the Hepatitis B Virus Subgenotypes in Brazil
Source: Viruses. 2019 Sep 15;11(9):860. doi: 10.3390/v11090860 (PMC6784006; doi:10.3390/v11090860)
Supplement: Supplementary file 1 [file viruses-11-00860-s001.pdf]

Table S1: Sequences used in HBV complete genome phylogenetic analyses

HBV/A

Genbank accession number	Subenotype*	Location
KF922437	A1	South Africa
KJ854707	A1	Brazil
AY233286	A2	South Africa
AB453979	A2	Japan
AY934764	A3 (*quasi*-A3)	Gambia
FN545825	A3 (*quasi*-A3)	Nigeria
GQ161813	A3 (*quasi*-A3)	Guinea
AY934764	A4 (*quasi*-A3)	Gambia
AM180623	A4 (*quasi*-A3)	Mali
FJ692554	A5 (*quasi*-A3)	Nigeria
FJ692556	A5 (*quasi*-A3)	Nigeria
GQ331047	A6 (new A4)	Belgium
GQ331048	A6 (new A4)	Belgium

HBV/B

Genbank accession number	Subenotype*	Location
AB900112	B1	Japan
AB981583	B2	Japan
JX869999	B2	Panama
MG372436	B2	China
KP341009	B3	Indonesia
KP341010	B4	Viet Nam
KP341012	B7	Indonesia
KP341013	B9	Indonesia
KP659255	B6	Indonesia

HBV/C

Genbank accession number	Subenotype*	Location
JX507214	C1	Panama
JQ801522	C1	Thailand
HQ622095	C2	Brazil
JN315779	C2	South Korea
KU679960	C4	Australia
KU695741	C3	Australia
KU695746	C3	Australia

KF873511	C4	Australia
KM999992	C5	Philippines
JN827415	C5	Thailand
KM999993	C6	Indonesia
EU670263	C6	Philippines

HBV/D

**Genbank accession number**	**Subenotype***	**Location**
KY382412	D1	Argentina
GU357846	D1	China
KP322601	D2	India
KU736925	D2	Sudan
KP090181	D3	Brazil
KP322602	D3	India
KC012652	D3	Argentina
KJ470898	D4	Brazil
GQ922004	D4	Canada
KP322603	D5	India
KF170740	D6	Sudan
KU736923	D7	Somalia
KP322604	D7	Tunisia

HBV/E

**Genbank accession number**	**Location**
AM494710	Central African Republic
KF849722	Angola
KT192626	Mexico

HBV/F

**Genbank accession number**	**Subenotype***	**Location**
KP995098	F1	Venezuela
KX264496	F1	Chile
KT896494	F2	Brazil
KP995115	F2	Venezuela
FJ589067	F3	Colombia
MH051987	F3	Venezuela
JX079937	F4	Argentina
KP995118	F4	Argentina

* according to the information available in Genbank

HBV/G

Genbank accession number	Location
GU565217	Netherlands
KF414679	Venezuela

HBV/H

Genbank accession number	Location
AB298362	Japan
HM117850	Mexico

HBV/I

Genbank accession number	Location
GU357844	China
KF214650	India

HBV/J

Genbank accession number	Location
AB486012	Japan
